# Teledermatology Exposes a Neglected Endemic: The Hidden Burden of Tinea Imbricata in Eastern Indonesia

**DOI:** 10.1111/ijd.70197

**Published:** 2025-12-09

**Authors:** Livia Ayuni, Maria Harianja, Messe R. Ataupah, Ruth D. Laiskodat, Jochem Buil, Claus Bøgh, Hardyanto Soebono, Marlous L. Grijsen

**Affiliations:** 1 Sumba Foundation, Wanokaka, Indonesia; 2 Province Health Office, East Nusa Tenggara, Kupang, Indonesia; 3 Department of Medical Microbiology, Radboudumc, Nijmegen, the Netherlands; 4 Radboudumc-CWZ Expertise Center for Mycology, Nijmegen, the Netherlands; 5 Department of Dermatology and Venereology, Universitas Gadjah Mada, Yogyakarta, Indonesia; 6 Center for Tropical Medicine, Faculty of Medicine, Public Health and Nursing, Universitas Gadjah Mada, Yogyakarta, Indonesia; 7 Oxford University Clinical Research Unit Indonesia, Faculty of Medicine Universitas Indonesia, Jakarta, Indonesia; 8 Centre for Tropical Medicine and Global Health, Nuffield Department of Medicine, University of Oxford, Oxford, UK

**Keywords:** Indonesia, neglected tropical diseases, skin-NTD, skin of color, teledermatology, tinea imbricata, *Trichophyton concentricum*

Tinea imbricata, also known as *Tokelau* in the Pacific, is a chronic, superficial fungal infection caused by *Trichophyton concentricum*, which is endemic in remote communities across Southeast Asia, the Pacific, and Latin America [1]. It typically presents as multiple concentric rings with fine scaling, and is often widespread [2]. Predisposing factors include socio-economic and environmental factors, like household crowding, high humidity, poor hygiene and possibly genetic predisposition [1, 2]. Tinea imbricata can significantly impact quality of life due to chronic pruritus and disfigurement. Diagnosis is primarily clinical, supported by direct microscopy and/or culture, although *T. concentricum* is slow-growing and difficult to culture. Oral antifungal therapy is challenging due to high recurrence rates, prolonged treatment requirements, and potential adverse effects [3].

Tinea imbricata has been sporadically reported in Indonesia [1]. We describe six cases identified through our teledermatology platform in Sumba, one of the most remote and impoverished islands in eastern Indonesia. Teledermatology serves as a valuable tool to enhance access to quality skin care in underserved settings; details of this programme have been published elsewhere [4].

Between 2021–2024, six individuals with tinea imbricata were identified, presenting with widespread pruritic, scaly lesions on the trunk and extremities (Figure 1a–f). Five were female; the median age was 62 years old (interquartile range [IQR] 57–64), with one 13-year-old girl. All individuals lived remotely in traditional bamboo houses with grass-thatched roofs and had limited access to clean water. The duration of infection ranged from 2–50 years. Three individuals reported that relatives experienced similar symptoms. Diagnoses were confirmed with direct microscopy of skin scrapings in 10% potassium hydroxide, revealing irregular, branched, septate fungal hyphae (Figure 1g).

Fungal cultures were performed at the Radboud University Medical Center, the Netherlands. Most were negative for dermatophytes due to overgrowth by non-relevant fungi. Nonetheless, a slow-growing dermatophyte cultured from Sabouraud Dextrose Agar supplemented with chloramphenicol and cycloheximide from one of the individuals was identified as *T. concentricum* via DNA sequencing of the ITS region of the rRNA gene. Susceptibility testing was performed using the EUCAST broth microdilution method for dermatophytes (E.Def 11.0). Minimum inhibitory concentrations (MICs) were 0.031 mg/L for itraconazole, 0.5 mg/L for miconazole, 1 mg/L for ciclopirox, and < 0.016 mg/L for terbinafine. Although no established breakpoints are available for interpretation, these MIC values are relatively low and do not suggest resistance.

Our first patient was treated with oral ketoconazole for three months, as no alternative antifungal drugs were available. The other patients were treated with griseofulvin for up to 1 year. As most patients were living far away from the clinic, clinical follow-up, drug renewal, and treatment adherence were challenging. All individuals demonstrated clinical improvement. However, so far, only one case has achieved complete remission.

This report describes the first case series of tinea imbricata on Sumba Island. The true burden remains largely unknown, although cases have previously been documented among indigenous communities in West Papua, Papua, Banten, Kalimantan, Sulawesi, and Lombok [1, 5]. Affected individuals often live in socioeconomically disadvantaged and geographically hard-to-reach communities with limited access to healthcare. Underreporting likely stems from limited awareness, inadequate training in skin disease management, and the absence of surveillance systems for accurate diagnosis and reporting.

Direct microscopy offers a practical, low-cost diagnostic tool facilitating timely treatment decisions, although it is not species-specific [6]. Laboratory infrastructure for fungal cultures or DNA sequencing is often lacking in resource-limited settings, where the burden of disease is highest, due to constraints in technical capacity and resources.

Previous randomized trials demonstrated that griseofulvin and terbinafine were more effective than itraconazole and fluconazole for tinea imbricata, sustaining remission for up to 8 weeks post-treatment [3, 5]. In contrast, our field experience showed less favorable outcomes, with most individuals achieving partial remission after one year. These suboptimal results likely reflect inconsistent drug availability, challenges in treatment adherence, and possible reinfection from undiagnosed contacts. Addressing the burden of skin diseases requires improved healthcare access, reliable drug supplies, and strengthened capacity among frontline providers. Further research is needed to explore host genetic susceptibility, characterize antifungal resistance patterns, and inform the development of more effective, context-specific treatment strategies for tinea imbricata.

## Figures and Tables

**Figure 1:**
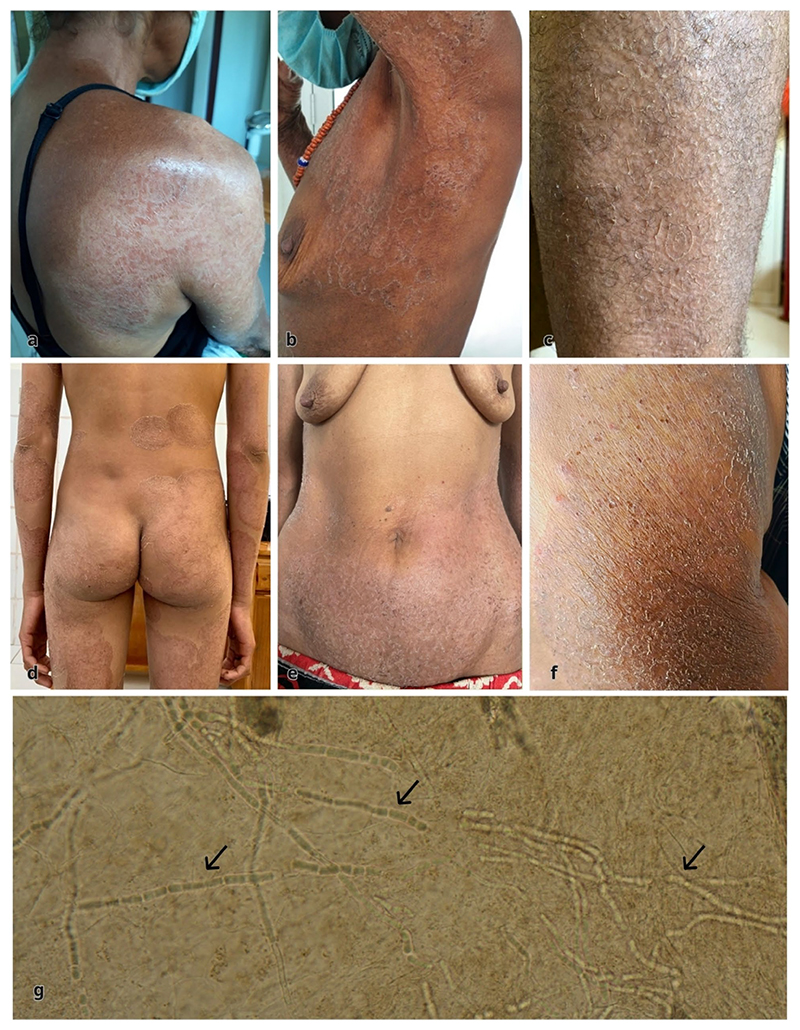
Clinical and microscopic findings of individuals affected by tinea imbricata in Sumba Island, eastern Indonesia. (a–f) Multiple polycyclic plaques consisting of concentric rings of scales affecting the limbs and trunks in six different individuals. (g) Direct microscopy using potassium hydroxide wet mount illustrating the characteristic branched, septate, and irregular hyphae (40× magnification).

## Data Availability

Data sharing is not applicable to this article as no datasets were generated or analysed during the current study.

## References

[1] Er YX, Lee SC, Than LTL (2022). Tinea Imbricata Among the Indigenous Communities: Current Global Epidemiology and Research Gaps Associated With Host Genetics and Skin Microbiota. Journal of Fungi.

[2] Hay RJ, Reid S, Talwat E, Macnamara K (1984). Endemic Tinea Imbricata: A Study on Goodenough Island, Papua New Guinea. Transactions of the Royal Society of Tropical Medicine and Hygiene.

[3] Wingfield AB, Fernandez-Obregon AC, Wignall FS, Greer D (2004). Treatment of Tinea Imbricate: A Randomized Clinical Trial Using Griseofulvin, Terbinafine, Itraconazole and Fluconazole. British Journal of Dermatology.

[4] Adella FJ, Ammah H, Siregar GO (2024). Teledermatology to Improve Access to and Quality of Skin Care in Eastern Indonesia. American Journal of Tropical Medicine and Hygiene.

[5] Budimulja U, Kuswadji K, Bramono S (1994). A Double-Blind, Randomized, Stratified Controlled Study of the Treatment of Tinea Imbricata With Oral Terbinafine or Itraconazole. British Journal of Dermatology.

[6] Siregar GO, Harianja M, Smith DJ (2025). Leveraging Malaria Microscopy Infrastructure to Diagnose Common and Neglected Skin Diseases Using Direct Microscopy in Sumba, Indonesia. Lancet Regional Health - Western Pacific.

